# Investigating the Structure of the Components of the PolyADP-Ribosylation System in Fusarium Fungi and Evaluating the Expression Dynamics of Its Key Genes

**DOI:** 10.32607/actanaturae.27450

**Published:** 2024

**Authors:** A. A. Stakheev, R. R. Kutukov, M. E. Taliansky, S. K. Zavriev

**Affiliations:** Shemyakin–Ovchinnikov Institute of Bioorganic Chemistry, Moscow, 117997 Russian Federation

**Keywords:** Fusarium, parylation, PARP, PARG, transcription regulation, mycotoxin, expression

## Abstract

Poly(ADP-ribose) polymerase (PARP) is the key enzyme in polyADP-ribosylation, one of the main post-translational modifications. This enzyme is abundant in eukaryotic organisms. However, information on the PARP structure and its functions in members of the Fungi kingdom is very limited. In this study, we performed a bioinformatic search for homologs of PARP and its antagonist, PARG, in the genomes of four* Fusarium *strains using their whole-genome sequences annotated and deposited in databases. The *F. graminearum* PH-1, *F. proliferatum *ET-1, and *F. oxysporum *Fo47 strains were shown to possess a single homolog of both PARP and PARG. In addition, the *F. oxysporum *f. sp. *lycopersici *strain 4287 contained four additional proteins comprising PARP catalytic domains whose structure was different from that of the remaining identified homologs. Partial nucleotide sequences encoding the catalytic domains of the PARP and PARG homologs were determined in 11 strains of 9 *Fusarium *species deposited in all-Russian collections, and the phylogenetic properties of the analyzed genes were evaluated. In the toxigenic *F. graminearum *strain, we demonstrated up-regulation of the gene encoding the PARP homolog upon culturing under conditions stimulating the production of the DON mycotoxin, as well as up-regulation of the gene encoding PARG at later stages of growth. These findings indirectly indicate involvement of the polyADP-ribosylation system in the regulation of the genes responsible for DON biosynthesis.

## INTRODUCTION

Plant diseases that are caused by phytopathogenic fungi are a significant problem for agriculture and the economy in all regions of the world [1, 2]. Members of some taxonomic groups of the Fungi kingdom, in particular the *Fusarium *genus, are able not only to infect agricultural crops, but also to produce toxic secondary metabolites – mycotoxins [3] – that inhibit protein synthesis, induce apoptotic processes, and exert hepatotoxic and immunosuppressive effects on mammals [4, 5, 6, 7]. The ability to synthesize trichothecene mycotoxins is a factor of fungal aggressiveness to host plants: mutant *F. graminearum *strains which had not produced deoxynivalenol (DON) were shown to be able to infect a plant, but the infection did not spread to other parts of the plant [8].

The biosynthesis of the main mycotoxin groups occurs in a similar manner: the key genes responsible for its various stages are grouped in clusters under the control of one or more regulatory factors [9, 10]. In turn, global transcription factors are mediators that regulate the expression of specific genes, depending on various environmental factors, such as temperature, humidity, pH, and nutrient availability [11, 12]. The key regulatory processes include histone protein modifications; in particular, poly(ADP-ribosyl)ation or parylation.

Parylation involves the transfer of several ADPribose residues from the NAD^+^ cofactor molecule to the target amino acid or nucleic acid, which changes the structure, function, and stability of the target molecule [13, 14]. The key enzyme responsible for the parylation reaction is poly(ADP-ribose) polymerase (PARP), which belongs to the poly(ADP-ribosyl) transferase family [15]. According to current concepts, enzymes of this family are structurally and functionally similar to the exotoxins of pathogenic bacteria, such as the diphtheria and cholera toxins [16, 17]. PARP is a fairly conservative enzyme; it is found in all eukaryotes, except yeast. The best characterized to date is human PARP1, which is a 116 kDa protein consisting of three main domains: the N-terminal DNA-binding domain comprising zinc finger structures, the central regulatory (BRCT) domain, and the C-terminal catalytic domain, which is the most conservative and characteristic domain of the proteins of the poly(ADP-ribosyl) transferase family [18, 19]. In addition, PARP1 homologs can contain the WGR (Trp–Gly–Arg) domain, transmembrane structures, and various regulatory sequences [20]. The spectrum of PARPregulated processes is quite wide: DNA repair, apoptosis, cell cycle regulation, gene transcription control, etc. [21, 22, 23, 24]. It is currently known that some eukaryotes contain several PARP copies; e.g., human cells contain, apart from PARP1, the homolog proteins PARP2 and PARP3, which are functionally similar to PARP1. In general, the human genome contains 17 genes encoding PARP family proteins [15, 25]. It should also be noted that parylation is a reversible modification: poly(ADP-ribose) glycohydrolase (PARG), a PARP antagonist, is responsible for the hydrolysis of ribose–ribose bonds and the cleavage of polymer chains [26, 27]. In the canonical version (human), PARG consists of a C-terminal catalytic macrodomain and an N-terminal regulatory part, although its structure may differ in other organisms [28].

It should be noted that, despite their versatility and high conservatism, information about the functions of the parylation system enzymes in Fungi, in particular *Ascomycota*, is quite limited. There are a number of studies indicating the involvement of PARP in apoptosis [29, 30], replicative aging [31], and the formation of asexual reproduction structures [32]. In this case, almost nothing is known about a potential role for PARP in the pathogenesis and biosynthesis of mycotoxins.

The aim of this study was to identify possible PARP and PARG homologs in members of the* Fusarium *genus using a bioinformatic search in open access databases and sequencing of *PARP *and *PARG* gene fragments in the strains of 9 *Fusarium *species common in Russia and neighboring countries. In addition, we compared the expression profiles of the PARP and PARG homologs in the *F. graminearum* strain capable of mycotoxin biosynthesis when grown on different media.

## EXPERIMENTAL

**Bioinformatic analysis**

To search for the PARP and PARG homologs, we selected four strains of *Fusarium *fungi whose whole genome structures have been annotated and deposited in online GenBank NCBI (https://www.ncbi.nlm.nih. gov/genbank/) and Fusarium oxysporum pangenome databases (http://www.fopgdb.site/): *F. graminearum* PH-1, *F. proliferatum *ET-1, *F. oxysporum *Fo47, and* F. oxysporum *f. sp. *lycopersici *4287. Amino acid sequences of the human PARP1 and PARG proteins and the *Aspergillus nidulans *PARP homolog (PrpA) and the nucleotide sequences of the corresponding genes were used as references. The homologs were searched using the BLAST algorithm [33]. Functional domains in the identified protein sequences were modeled using the InterPro online service (https://www.ebi.ac.uk/ interpro/).

**Fungal strains**

In this study, we used 11 strains of 9 species of the* Fusarium *genus from the collections of the Federal Research Center of Nutrition and Biotechnology (FRCNB), the All-Russian Research Institute of Plant Protection (VIZR), and the Pushchino Scientific Center for Biological Research (All-Russian Collection of Microorganisms, VKM). The list of strains, with their geographical origin, host plant species, collection years, and collection affiliation, is provided in *Table 1*. The cultures were grown on potato sucrose agar at 25°C for 7 days.

**Table 1:** Fungal strains used in the study

Strain	Species	Collection	Source of isolation	Geographical origin	Year
MFG 58918	F. graminearum	VIZR	Wheat grain	Krasnodar Region	2016
ION-17-9/8	F. graminearum	FRCNB	Wheat grain	Moscow Region	2017
MFG 96801	F. oxysporum	VIZR	Wheat grain	North Ossetia	2007
F-840	F. oxysporum	VKM	Unknown	Germany	Unknown
MFG 58242	F. venenatum	VIZR	Unknown	Germany	2008
ION-3/4	F. coffeatum	FRCNB	Wheat grain	Tula Region	2014
F-3495	F. redolens	VKM	Barley grain	Moscow Region	Unknown
F-206	F. verticillioides	VKM	Tobacco plant	Krasnodar Region	Unknown
F-446	F. fujikuroi	VKM	Rice grain	Japan	Unknown
MFG 61701	F. poae	VIZR	Wheat grain	Saratov Region	2010
F-3951	F. solani	VKM	Soil	Moscow Region	Unknown

**Isolation of nucleic acids**

DNA was isolated according to the previously reported technique [34]. RNA was isolated from liquid cultures of the *F. graminearum *MFG 58918 fungus using a RNeasy Plant Mini Kit (Qiagen, Germany) according to the manufacturer’s protocol. The concentration and quality of the nucleic acids were assessed using a NanoVue spectrophotometer (GE HealthCare, USA), a Qubit fluorometer (Thermo Scientific, USA), and electrophoresis in 1% agarose gel.

**Primer design, PCR, and sequencing of amplification products**

The primers for PCR and sequencing were designed using the ClustalW algorithm [35]. The physicochemical properties of the primers were assessed using the Oligo 6.71 software.

The structures of the primers and probes used to assess the relative expression of the reference gene* TEF1α *and the trichodiene synthase gene *TRI5 *had been published previously [36].

PCR was performed on a Tertsik thermal cycler (DNA-Technology, Russia) using the following amplification programs:

Program 1 (PARPF-R primers): 93°C, 90 s (1 cycle); 93°C, 1 s; 55°C, 5 s; 72°C, 5 s (40 cycles).

Program 2 (PARGF-R primers): 93°C, 90 s (1 cycle); 93°C, 10 s; 60°C, 15 s; 72°C, 10 s (40 cycles).

Quantitative PCR for assessing relative transcript abundances was performed on a DT-96 detecting thermal cycler (DNA-Technology) using Program 2. Transcript accumulation was assessed using the QGene software [37].

The PCR products were cloned using a Quick-TA kit (Eurogen, Russia) according to the manufacturer’s protocol. DNA fragments were sequenced by the Sanger technique at Evrogen JSC using an ABI PRISM BigDye Terminator v. 3.1 reagent kit, followed by analysis of the reaction products on an ABI PRISM 3730 Applied Biosystems automatic sequencer.

The resulting nucleotide sequences were deposited in the NCBI GenBank database (accession numbers PQ040409–PQ040429).

**Phylogenetic analysis**

Multiple alignment and the phylogenetic analysis of the amino acid and nucleotide sequences, as well as assessment of their phylogenetic properties, were performed using the MEGA7 software package [38]. Phylogenetic trees were constructed using the maximum likelihood (ML) method and the Jones–Taylor– Thornton (for amino acids, [39]) and GTR+G (for nucleotides, [40]) models. The robustness of the phylogenetic tree topologies was confirmed by bootstrap analysis (1,000 replicates).

**Evaluation of the accumulation of the DON mycotoxin produced by *F. graminearum *in the liquid culture**

To evaluate the accumulation of mycotoxin deoxynivalenol (DON), the *F. graminearum *MFG 58918 strain was cultured in two media of different compositions: a potato sucrose broth and the MYRO medium [41]. For this purpose, the liquid media (10 mL) were inoculated with 100 μL of a *F. graminearum *MFG 58918 conidium suspension and incubated at 25°C for 7 days. Material for the analysis of the relative expression of the target genes was collected every 24 h, from days 2 to 7. Samples for the analysis of mycotoxin levels were collected on days 4 and 6 of culture.

The mycotoxin content in the media was analyzed by an immunochemical express method using DONSENSOR test kits (Unisensor, Belgium) according to the manufacturer’s protocol.

## RESULTS

**Fig. 1:**
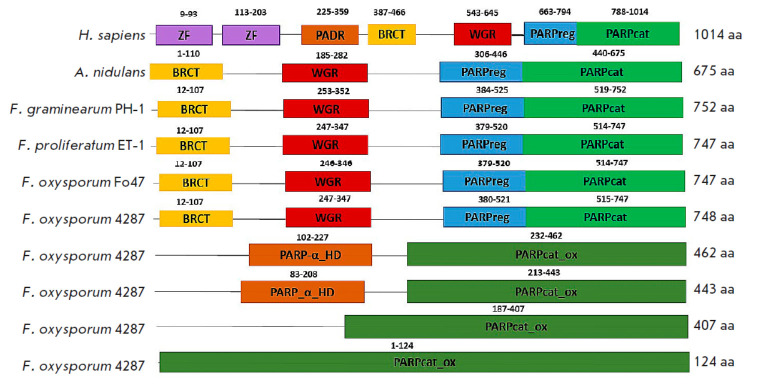
Schematic representation of the main functional domains of human PARP1, the *A. nidulans *PrpA, and PARP homologs of four *Fusarium *strains. The range of amino acids in each domain is indicated above the corresponding domain. ZF is a ‘zinc finger’ structure

**Search for PARP and PARG homologs**

**Fig. 2:**
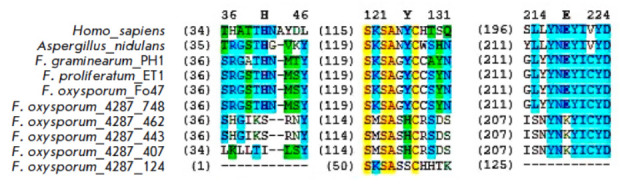
Fragments of amino acid sequence alignment of human PARP1, the *A. nidulans *PrpA, and PARP homologs of four *Fusarium *strains, which contain amino acid residues constituting the H–Y–E motif. Key amino acids are shown in bold

A search performed using the BLASTp algorithm revealed that the genomes of *F. graminearum *PH-1,* F. proliferatum *ET-1, and the *F. oxysporum *Fo47 and 4287 strains contained one open reading frame, and that their translation products were presumably human PARP1 and *A. nidulans *PrpA homologs. The identified proteins consisted of 747–752 amino acid residues and contained four putative domains: BRCT, WGR, regulatory, and catalytic. Unlike human PARP1, they lacked the N-terminal domain, which contains zinc finger structural motifs, and the pADR subdomain (*Fig. 1*). In general, the structure of the identified homologs corresponded to that of the previously characterized PrpA from *A. nidulans* [32]. Analysis of amino acid sequence similarities revealed that the most conserved part of all analyzed proteins was the catalytic domain: for example, the identity of the catalytic domain sequences of human PAPR1 and its homolog in *F. graminearum* PH-1 was 43.9% and the identity of the sequences of the four analyzed *Fusarium *strains was 85.1%. Meanwhile, comparison of the whole PARP sequences showed a degree of identity of 24.5% between the humans and *F. graminearum *and 76.6% among the* Fusarium *strains. Also, an amino acid motif – histi- dine–tyrosine–glutamic acid (H–Y–E, *Fig. 2*) – was revealed in the homolog structures, which is, according to current concepts, key for the catalytic function of PARP.

**Fig. 3:**
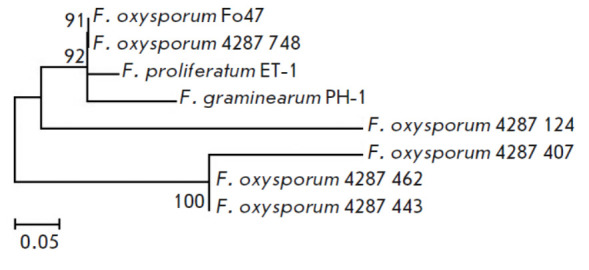
The phylogenetic tree constructed based on alignment of the amino acid sequences of the catalytic domains of the PARP homologs identified in the genomes of four* Fusarium *strains using the maximum likelihood method. Bootstrap values of >50% (1,000 replicates) are shown

In addition, a search for PARP homologs using only the amino acid sequences of the PARP1 and PrpA catalytic domains as a query revealed four more open reading frames in the genome of the *F. oxysporum* f. sp. *lycopersici *4287 strain, whose translation products contained PARP catalytic domains (*Fig. 1*). Two of them were the 443 aa (FoxPARP443, GenBank accession number XP_018253699) and 462 aa (FoxPARP462, XP_018251710) proteins containing alpha-helical (PARP αHD) subdomains at the N-terminus of their catalytic domains. Another predicted 407 aa homolog (FoxPARP407, XP_018251711) contained only the catalytic domain, without the αHD subdomain. The fourth homolog was a short 124 aa protein (FoxPARP124, XP_018251751) the entire structure of which was characterized as a catalytic domain. It is interesting to note that the genes encoding the four “additional” PARPs are located on different chromosomes (chromosome 3: FoxPARP462, FoxPARP407, and FoxPARP124; chromosome 6: FoxPARP443; the gene encoding the “main” PARP is located on chromosome 4). The structures of these homologs differed significantly from each other and from the “main” PARP homologs of *F. oxysporum *and other species. The degrees of identity of the catalytic domain structures of FoxPARP443 and FoxPARP407, FoxPARP443 and FoxPARP124, FoxPARP443 and “main” PARP of *F. oxysporum *f. sp. *lycopersici*, and FoxPARP443 and human PARP1 were 67%, 52.8%, 57.2%, and 31.2%, respectively. These results were confirmed by data from a phylogenetic analysis of the amino acid sequences of the catalytic domains (*Fig. 3*). The dendrogram shows two clusters supported by bootstrap values of 92 and 100%: the first included the “main” domains of the four analyzed strains, and the second included the catalytic domains of the FoxPARP462, FoxPARP443, and FoxPARP407 proteins. The FoxPARP124 protein domain formed a separate branch in an intermediate position between the two clusters. In addition, neither the catalytic H–Y–E motif nor any other arginine–serine–glutamic acid (R–S–E) motif typical of the family of poly(ADP-ribosyl) transferases was revealed in the structure of the catalytic domains of the “additional” PARPs.

**Fig. 4:**
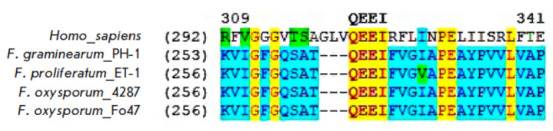
A fragment of amino acid sequence alignment of the human PARG and PARG homologs of four *Fusarium* strains, which contains the Q–E–E–I motif (shown in bold)

PARG homologs were searched using human PARG as a reference. One open reading frame was identified in each of the analyzed genomes. The translated proteins consisted of 443–476 aa and had a common structure, including an N-terminal α-helical and a C-terminal catalytic domain. In all the structures of the discovered homologs, a glutamine–glutamic acid– glutamic acid–isoleucine (Q–E–E–I) motif was identified, which is considered essential for the functional activity of the enzyme (*Fig. 4*). In this case, the analysis of amino acid sequence similarities revealed that PARG was a less conservative protein than PARP: the level of identity of PARG from the four analyzed* Fusarium *strains was 60.5%, and that of the *Fusarium* and human PARG homologs was only 17.6%.

**Primer design, sequencing, and phylogenetic analysis of the fragments of the genes encoding the PARP and PARG homologs**

**Fig. 5:**
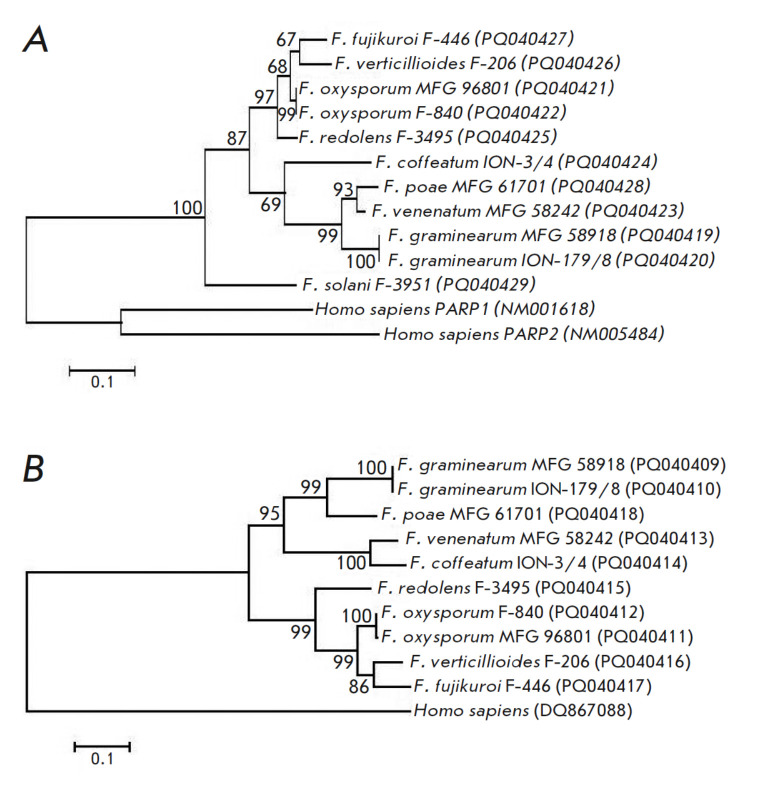
Phylogenetic trees constructed based on nucleotide sequence alignments of PARP (*A*) and PARG (*B*) of eleven *Fusarium *strains from all-Russian collections using the maximum likelihood method. Bootstrap values of >50% (1,000 replicates) are shown. The GenBank accession number of each strain studied is shown in brackets

On the basis of alignment of the nucleotide sequences of the genes encoding the PARP and PARG homologs identified in the bioinformatic analysis, we constructed universal primers for the sequencing of their fragments. The sequence encoding the PARP catalytic domain, the *PARG *gene fragment encoding the C-terminus of the α-helical domain, the N-terminus of the catalytic domain, and the region connecting them were selected as targets. The structures of the developed primers were as follows: PARP: forward primer PARPF (5’-ATCCTCTYGATCGHCARTT-3’), reverse primer PARPR (5’-GHAGSAGRTAVCGBAGCTTG-3’); PARG: forward primer PARGF (5’-GGYAAAATHCCATTYTGGCC- 3’), reverse primer PARGR (5’-AGACVACGACDGCHCCTCCTT-3’). We found that the pair of PARPF–R primers amplified the DNA fragments of all the 11 strains selected for the study (*Table 1*) and that the pair of PARGF–R primers amplified all, except for the DNA of the *F. solani *F-3951 strain. The sizes of the amplified fragments were 611 bp for PARP and 596–611 bp for PARG. Analysis of the phylogenetic characteristics of the sequenced fragments confirmed the suggestion that PARG was less conservative than PARP: the fragment of the gene encoding the PARP homolog contained 58.7% conservative, 41.3% variable, and 25.8% parsimony informative positions, whereas the fragment of the gene encoding the PARG homolog contained 42.8% conservative, 57.1% variable, and 42.4% parsimony informative positions. The phylogenetic tree constructed based on the analysis of the fragments of the gene encoding the PARP homolog (*Fig. 5A*) contained two main clusters, one of which contained species capable of synthesizing trichothecene toxins (*F. graminearum*,* F. poae*, *F. venenatum*, *F. coffeatum*; bootstrap support, 69%), and the other that included species producing other groups of toxins (*F. fujikuroi*, *F. verticillioides*,* F. oxysporum*, *F. redolens*; bootstrap support, 96%). The *F. solani *F-3951 strain formed a separate branch; it should be noted that the entire group of* Fusarium *strains formed a single, large cluster with a bootstrap support value of 100%. A similar picture was observed in the analysis of the phylogenetic tree constructed based on a comparison of fragments of the gene encoding the PARG homolog (*Fig. 5B*), with the difference being that the bootstrap support value for clusters containing trichothecene-producing and non-producing species was higher (94 and 99%, respectively). In addition, there were topological differences related to the fact that *F. venenatum *and *F. coffeatum* formed a separate subcluster with a bootstrap support value of 100%.

**Analysis of DON mycotoxin accumulation and the expression dynamics of genes encoding the PARP and PARG homologs during fungal growth on different media**

**Fig. 6:**
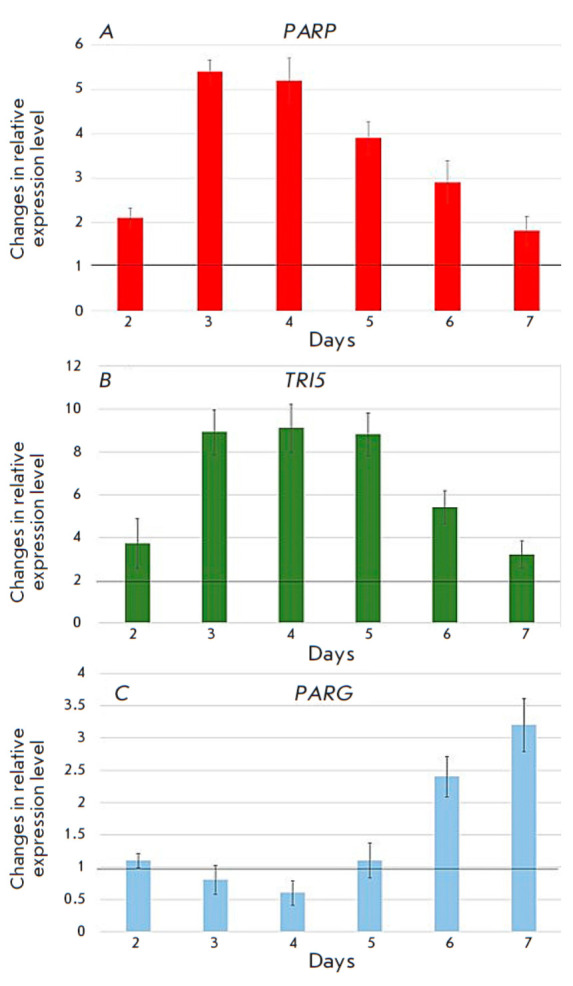
Estimation of the relative expression levels of the* FgPARP *(*A*), *TRI5 *(*B*), and *FgPARG *(*C*) genes on days 2 to 7 of culture on the MYRO medium compared to the control (PSB, set as 1 unit)

To compare toxin formation levels, the *F. graminearum* MFG 58918 strain was cultured on a potato sucrose broth (PSB), which is considered favorable for the growth of fungal biomass, and a MYRO medium, which stimulates mycotoxin biosynthesis (in particular DON). Analysis of DON accumulation in the MYRO medium showed that its concentration was 30.6 mg/L of the medium on day 4 and 39.9 mg/L of the medium on day 6. In this case, in cultures grown on PSB, DON was not detected at any of those time points. In addition to the genes encoding the PARP (*FgPARP*) and PARG (*FgPARG*) homologs, analysis of the relative expression dynamics also included the* TRI5 *gene encoding trichodiene synthetase, a key enzyme in the biosynthesis of trichothecene toxins. We found that the relative expression of both *TRI5* and *FgPARP *upon growth on the MYRO medium exceeded that in the control (PSB) at each time point. The relative expression of the *FgPARP *gene was maximal on day 3 of growth (5.4-fold higher than in the control, *Fig. 6A*), and that of the *TRI5 *gene was maximal on day 4 (8.6-fold higher than in the control, *Fig. 6B*). However, the relative expression levels of both genes decreased on days 6 to 7. Relative expression of the *FgPARG *gene on days 2–5 of growth on the MYRO medium was virtually identical to that in the control culture, but it increased on days 6 to 7 (2.3- and 3.2-fold higher, respectively, than in the control, *Fig. 6B*).

## DISCUSSION

PARP is an enzyme that is involved in many important cellular processes and is found in organisms from different taxonomic groups. The importance of studying PARP and the parylation system is generally determined by its possible practical significance, in particular the use of this enzyme as a target for drugs and agents against pathogens, including those causing plant diseases [42, 43, 44, 45, 46]. However, information on the structure and functions of parylation system components in fungi is currently extremely scarce. There is data indicating that *Saccharomyces cerevisiae *and* Schizosaccharomyces pombe *yeasts lack PARP homologs [47]. On the other hand, the toxigenic fungus* A. nidulans *contains a human PARP1 homolog (PrpA) that is involved in DNA repair and asexual development [32]. It has been shown that a PARP homolog from *F. pseudograminearum *participates in apoptosis [30], and that treatment of *F. oxysporum *with an inhibitor of the biosynthesis of NAD^+^, the main PARP substrate, reduces the growth and pathogenicity of the fungus [48]. In this case, one of the most interesting questions is the potential involvement of PARP and its homologs in the regulation of mycotoxin biosynthesis, in particular fusariotoxins. Currently, histone modifications and chromatin structure changes are considered to be some of the main factors influencing the activity of genes and biosynthetic clusters in general [49, 50], but the role of parylation in these processes in fungi is unknown.

In the present work, we searched for homologs of PARP and its antagonist PARG using databases containing whole genome structures of fungi of the* Fusarium *genus with predicted translation products. In each of the four studied genomes, we found approximately 750 aa PARP1 and PrpA homologs possessing a universal structure: BRCT and WGR domains at the N-terminus and regulatory and catalytic domains at the C-terminus. Unlike human PARP1, the fungal enzymes lacked a N-terminal regulatory domain containing the zinc finger motifs responsible for the search for and recognition of DNA damage and PARP binding to it, which may be an indication of their greater relation to human PARP2, which also lacks these structures [15], than to PARP1. In this regard, the question of how fungal PARPs bind to DNA and perform repair remains open. Possible alternatives to zinc fingers may be involvement of the WGR domain [20] or the use of an intermediary protein in the binding process. One of the most interesting results of the study was the identification, in addition to the “main” PARP homolog, of four proteins containing the catalytic domain, but lacking the WGR and BRCT domains in the *F. oxysporum *f. sp. *lycopersici* 4287 strain. Further search in the database using the BLASTp algorithm revealed that proteins containing the catalytic domain and structurally similar to the “additional” PARPs found in strain 4287 were also present in other *Fusarium *strains, primarily in *F. oxysporum*. This species is very plastic; in addition to the main (core) chromosomes, its genome can also contain additional chromosomes that often carry genes associated with pathogenicity and specificity to specific host plants [51, 52]. Probably, fungi have acquired “additional” proteins containing PARP catalytic domains independently of the “main” ones through horizontal gene transfer, whose indirect indication may be the presence of similar proteins in some plants, such as alfalfa or wheat. However, “additional” PARPs cannot completely duplicate the functions of the “main” ones due to the lack of a number of functionally significant domains in their structure. However, this does not equate to a functional inferiority of these proteins because what causes changes in the chromatin structure and nucleosome rearrangements may not be associated with the enzymatic activity of PARP [53].

The functional activity of the identified PARP1 homologs was indirectly confirmed by a search for amino acid motifs, which are believed to play a key role in the catalytic action of enzymes. This result is important from an evolutionary point of view: according to current concepts, bacterial exotoxins, such as the diphtheria and cholera toxins, are precursors of parylation system enzymes in eukaryotes. They are able to attach monoADP-ribose residues (mono(ADP-ribosyl) ation or marylation) to the proteins of the host organism, thereby exerting a negative effect on physiological and biochemical processes [17, 42]. The key catalytic motif of the diphtheria toxin is H–Y–E, and that of the cholera toxin is R–S–E. Accordingly, the families of eukaryotic poly(ADP-ribosyl) transferases derived from these two toxins differ in the presence of one of these motifs [54]. In this study, we showed that the catalytic centers of PARP homologs in *Fusarium* fungi contain the catalytic motif H–Y–E and, thus, may be attributed to the family of enzymes derived from the diphtheria toxin. In this case, the “additional” PARPs of strain 4287 did not contain any of these catalytic motifs, which also indicates the independent nature of their origin. Also, we found that each of the studied *Fusarium *strains contained one PARG homolog of the classical structure with the key catalytic motif Q–E–E–I.

Another objective of the study was to augment available information on the structure and polymorphism of genes encoding the PARP and PARG homologs in *Fusarium *genus fungi. At present, databases contain only single records of nucleotide sequences characterized as genes encoding components of the parylation system. Universal primers were developed, and sequencing and phylogenetic analysis of fragments of the corresponding genes were performed in strains of nine species of the* Fusarium *genus available in all-Russian collections. The fragment encoding the catalytic domain of the PARP homolog was shown to be more conserved than the gene fragment encoding the PARG homolog. Interestingly, the topologies of phylogenetic trees constructed based on a comparison of the structures of these two genes also differed slightly. According to the results of the analysis of PARG homologs, the* F. coffeatum *and *F. venenatum *species form a separate subcluster supported by a bootstrap value of 100%. This result looks unusual because, despite the similarity of the toxin profiles (the ability to synthesize type A trichothecene toxins), these two species belong to different species complexes (*F. coffeatum* belongs to the Fusarium incarnatum-equiseti species complex, and *F. venenatum *belongs to the F. sambucinum species complex). In this case, the topology of the phylogenetic tree constructed based on a comparison of the nucleotide sequences of the gene fragment encoding the PARP homolog had a more classical appearance: *F. venenatum *formed a subcluster with a closely related *F. poae*, and *F. coffeatum* formed a separate branch.

In this study, we assessed for the first time the expression dynamics of genes encoding the PARP and PARG homologs in toxigenic *F. graminearum *under conditions favorable for toxin synthesis and on a medium where the toxin was not produced. An increase in the relative expression level of *FgPARP *was shown to correlate with toxin accumulation and increased expression of *TRI5*, a key gene of the biosynthetic cascade. At later stages of culture (days 6 to 7), we observed an increase in the expression level of the* FgPARG *gene, which is probably associated with deparylation and a decrease in toxin biosynthesis. It should be noted that these results only indirectly suggest a relationship between the activity of genes encoding the proteins of the parylation system and toxin biosynthesis. For more reliable data proving these facts, further research is needed: in particular, the production of mutant strains and/or suppression of the expression of target genes using other approaches without genetic transformation; e.g., RNA interference.

## CONCLUSION

Based on the results of this study, we have significantly expanded and systematized the amount of information about the presence of components of the parylation system in phytopathogenic fungi of the* Fusarium *genus, as well as the structure, polymorphism, and activity of the corresponding genes. Our findings form the basis for further research into the role played by parylation in the vital activity of fungi, as well as the possible development of new approaches to combating these pathogens.

## Abbreviations

PARP: poly(ADP-ribose) polymerase
PARG: poly(ADP-ribose) glycohydrolase
PCR: polymerase chain reaction
DON: deoxynivalenol
bp: base pair
aa: amino acid
